# Endocytosed nanoparticles hold endosomes and stimulate binucleated cells formation

**DOI:** 10.1186/s12989-016-0173-1

**Published:** 2016-11-29

**Authors:** Lin Xia, Weihong Gu, Mingyi Zhang, Ya-Nan Chang, Kui Chen, Xue Bai, Lai Yu, Juan Li, Shan Li, Gengmei Xing

**Affiliations:** 1 CAS Key Laboratory for Biomedical Effects of Nanomaterial & Nanosafety, Institute of High Energy Physics, Chinese Academy of Science (CAS), Beijing, 100049 China; 2 School of Bioscience & Bioengineering, South China University of Technology (SCUT), Guangzhou, 510006 China

**Keywords:** Nanoparticles, Endocytic, Endosome, Large vesicle-like structures, Binucleated cell

## Abstract

**Background:**

Nanotechnology developed rapidly in cellular diagnosis and treatment, the endocytic system was an important pathway for targeting cell. In the research of developing macrophages as drug carriers or important therapeutic targets, an interesting phenomenon, internalized nanoparticles induced to form binucleated macrophages, was found although the particles dose did not cause obvious cytotoxicity.

**Results:**

Under 25 μg/ml, internalized 30 nm polystyrene beads(30 nm Ps nanoparticles) induced the formation of binucleated macrophages when they entered into endosomes via the endocytic pathway. These internalized 30 nm Ps nanoparticles (25 μg/ml) and 30 nm Au-NPs (1.575 ng/ml) also induced markedly rise of binucleated cell rates in A549, HePG-2 and HCT116. This endosome, aggregated anionic polystyrene particles were dispersed and bound on inner membrane, was induced to form a large vesicle-like structure (LVLS). This phenomenon blocked transport of the particles from the endosome to lysosome and therefore restricted endosomal membrane trafficking through the transport vesicles. Early endosome antigen-1 and Ras-related protein-11 expressions were upregulated; however, the localized distributions of these pivotal proteins were altered. We hypothesized that these LVLS were held by the internalized and dispersed particles decreasing the amount of cell membrane available to support the completion of cytokinesis. In addition, altered distributions of pivotal proteins prevented transfer vesicles from fusion and hampered the separation of daughter cells.

**Conclusions:**

30 nm Ps nanoparticles induced formation of LVLS, blocked the vesicle transport in endocytic system and the distributions of regular proteins required in cytokinesis which led to binucleated cells of macrophages. Markedly raised binucleated rate was also observed in human lung adenocarcinoma epithelial cell line(A549), human hepatoma cell line(HePG-2) and human colorectal cancer cell line(HCT116) treated by 30 nm Ps nanoparticles and Au-NPs.

**Electronic supplementary material:**

The online version of this article (doi:10.1186/s12989-016-0173-1) contains supplementary material, which is available to authorized users.

## Background

Nanotechnology developments are facilitating the use of engineered particles to diagnose and treat diseases on the cellular level. Many researchers have investigated various nanoparticle properties including surface charge, size, shape, and rigidity, while others have studied how to control the interaction of particles with the cell membrane to allow endocytic transport into target cells [1–3]. While many reports have confirmed the biocompatibility of nanoparticles, one major question is whether they will interfere in the native physiological functions of the endocytic system, such as membrane trafficking.

The eukaryotic endocytic system consists of pleiotropic intracellular organelles including endosomes. Recent studies have revealed that the endosome, which was a membrane pool, participated in membrane trafficking to maintain membrane cellular equilibrium. Disruption of this balance would affect cell physiologic functions and even result in cytokinesis failure [4]. Endosomes are pivotal organelles in the endocytic pathway, they transport cargoes to the lysosome and serve as the primary site for the returning and transport of plasmalemma and protein sorting [5]. Previous studies have shown that cargo proteins mediated the engulfment vesicles passing through the endosomes [6]. Nanoparticles enter cells by different endocytic pathways and are transported into endosomes via endosomal-lysosomal pathway. It remains unclear whether nanoparticles with certain properties could impact the physiological functions of endosomes.

A growing number of researchers have shown interest in developing macrophages as drug carriers or important therapeutic targets with increasing understandings of macrophage’s biological roles in disease. Endocytosis is a fundamental function of macrophages that facilitate the destruction and subsequent degradation of ingested microbes or allogenic materials. To perform this function, macrophages must rapidly replenish their cell surface membrane from intracellular membrane pools constructed with recycling endosomes. Fielding and Wilson groups have demonstrated that endosomes are also involved in mediating cellular cytokinesis and have also shown that sufficient membrane and pivotal proteins have to be available to allow the division of a mother cell into two daughter cells. We found that when internalized 30 nm Ps nanoparticles entered endosomes in macrophages, it restricted the recycling of endosomes back to the cell surface and binucleated cells become visible under the microscope. Although the particles aren’t necessarily toxic, these binucleated cells are genetically unstable. Therefore, we investigated how nanoparticles induced formation of binucleated cells following their endocytosis.

## Methods

### Cell culture

The murine macrophage cell line RAW 264.7, human cancer cell lines A549, HePG-2 and HCT116 were purchased from the Cancer Hospital at the Chinese Academy of Medical Sciences (Beijing, China). The murine macrophage cell line RAW264.7 was cultured in Dulbecco’s minimal essential media (DMEM)/F-12 (Hyclone Laboratories, Logan, UT) supplemented with 10% fetal bovine serum (FBS) in a humidified atmosphere of 5% CO2 at 37 °C. Human cancer cell lines A549, HePG-2, HCT116 cell lines were cultured in Dulbecco’s minimal essential media (DMEM)/HIGH GlUCOSE (Hyclone Laboratories, Logan, UT) supplemented with 10% fetal bovine serum (FBS) in a humidified atmosphere of 5% CO_2_ at 37 °C.

### Nanoparticle preparation

30 nm Ps nanoparticles labelled with FITC were purchased from Sigma (St. Louis, MO) and subsequently characterized using a scanning electron microscope (SEM; model S-4800; Hitachi, Tokyo, Japan). The NicompTM 380 ZLS particle size/zeta potential analyzer (Agilent Technologies, Santa Clara, CA) was used to determine the zeta potential of the particles with or without sonication. The measurements were performed in complete cell culture media at pH 7.4. Synthesis of 30 nm Au nanoparticles (Au-NPs) was according to citrate reduction method [7] and subsequently characterizing of the nanoparticles used SEM and DLS.

### Measure of cell viability

200 ul RAW 264.7 cells suspension (1 × 10^4^ cells/ml) were placed in every well of 96-well culture plate and cultured for 24 h, then the medium was respectively replaced with fresh medium containing 5, 25, and 50 μg/ml 30 nm Ps nanoparticles with 10-well replicates. After 24 or 48 h, cell viability was measured using Cell Counting Kit (CCK-8) ((Dojindo Laboratories, Japan) according to the manufacturer’s instruction. Following, viabilities of A549, HePG-2 and HCT116 cells treated by 30 nm Ps nanoparticles (25 μg/ml) and 30 nm Au-NPs (0.7875,1.575,3.15 ng/ml) was respectively measured.

### Cellular uptake of particles

A 1-ml RAW264.7 cells suspension (1 × 10^5^ cells/ml) was added to a confocal petri dish and cultured for 24 h, then the medium was replaced with fresh medium containing 25 μg/ml 30 nm Ps nanoparticles. Cells were cultured at 37 °C in a humidified atmosphere of 5% CO_2_ for 1 h, then were washed with PBS and fixed with 4% paraformaldehyde for 30 min at room temperature. Treated cells were observed and imaged with a confocal laser scanning microscope (Perkin-Elmer spinning disc confocal microscope [Waltham, MA] with a Nikon microscope [Nikon, Tokyo, Japan]) at a magnification of 400×. In the second group, the cells were cultured in particle-containing medium at 37 °C for 24 h. A movie of cells treated by particles for 11 h was taken in the confocal laser scanning microscope over a 60-min period (1 frame/min) at a magnification of 400 ×.

### Flow cytometer assay and cell counting

RAW 264.7 cells were cultured in medium with 25 μg/ml 30 nm Ps nanoparticles for 24 h and then were divided into two groups. Fist group, cells were digested in 2 ml trypsin. After 2 min, 2 ml fresh DMEM/F-12 supplement with 10% fetal bovine serum (FBS) was added to end the treatment, and the medium containing the treated cells was centrifuged (1200 rpm, 3 min). The cells were collected and washed with PBS (0.01 M, pH 7.2–7.4) three times, and then cold alcohol was added for 1–2 h to fix the cells. Fixed cells were collected through centrifugation (1200 rpm, 3 min), and propidium iodide was added to the tubes for 30 min to dye the DNA. The DNA quantities of treated cells were detected with a flow cytometer (Accuri C6 BD, Ann Arbor, MI) in the FL-2H channel. Second group, cells were washed with PBS and fixed with 4% paraformaldehyde for 30 min at room temperature. Treated and control cells were observed with a confocal microscope [Nikon Ti-E imaging system, Tokyo, Japan] and the binucleated macrophages were counted in ten stochastic views. The rate of binucleated cells was calculated and the cell was imaged at a magnification of 400 ×.

A549, HePG-2 and HCT116 cells were respectively cultured in medium with 25 μg/ml Ps nanoparticles for 48 h(data not shown), and then cells were washed with PBS and fixed with 4% paraformaldehyde for 30 min at room temperature. Treated and control cells were respectively observed with a confocal microscope and the binucleated cells were counted in ten stochastic views. The number of counted cells was more than 300 for each kind of cell line. The rate of binucleated cells was calculated. Image of cell was at a magnification of 400×. In other treatment, these cells were respectively cultured in medium with 1.575 ng/ml Au-NPs for 24 h, and then followed above.

### Immunofluorescent imaging

RAW 264.7 cells were cultured in confocal petri dishes for 24 h, then the medium was replaced with fresh medium containing 25 μg/ml 30 nm Ps nanoparticles and the cells were cultured at 37 °C in a humidified atmosphere of 5% CO2 for 10, 30 and 50 min. Treated cells were washed with 0.01 M phosphate-buffered saline (PBS) and fixed with 4% paraformaldehyde for 30 min at room temperature. Then paraformaldehyde was aspirated, and the cells were rinsed in PBS three times. Primary antibodies against early endosomal antigen 1 (EEA1) (CST, Danvers, MA), Ras-related protein-7 (Rab7) (Abcam, Cambridge, UK), lysosomal-associated membrane protein 1 (LAMP-1) (Abcam, Cambridge, UK), and Ras-related protein-11 (Rab11) (Abcam, Cambridge, UK) were used to label EEA1, Rab7, LAMP-1, and Rab11 proteins respectively. Specifically, Rab11 and EEA1 were used to label the cell of division in the late stages. For these experiments, cells were cultured in particle-containing medium for 1 h, were then transferred into fresh medium for 12 h. The dilutions and incubation times were according to the antibody manuals provided by the manufacturers. After the primary antibody incubation, cells were incubated with a secondary antibody with rhodamine (Abcam, Cambridge, UK) to identify cellular distributions of EEA1-, Rab7-, LAMP-1-, and Rab11-positive vesicles. Hoechst 33342 (Thermal fisher scientific, Waltham, MA,) was used to label the nuclei. Prepared petri dishes were observed and imaged with a confocal fluorescent microscope (Nikon Ti-E imaging system, Tokyo, Japan) at a magnification of 400×. Redistributions of the particles (green), EEA1(red), Rab7(red) and LAMP-1(red) at 10, 30 and 50 min in the cells were probed by imaging of confocal fluorescence microscope.

### Transferrin imaging

RAW 264.7 cells adherent to round coverslips were pre-incubated with 25 μg/ml 30 nm Ps nanoparticles in DMEM/F-12 for 12 h at 37 °C. The medium was aspirated and discarded, the cells were washed three times with PBS followed by incubation on ice for 20 min in DMEM/F-12 medium containing 20 mM glucose and 1% bovine serum albumin (BSA). Next, we added transferrin-Alexa Fluor 555 (Thermo Fisher Scientific, Waltham, MA) to the medium at a final concentration of 2.5% (volume/volume) and incubated at 37 °C for 20 min. Afterward, cells were washed with fresh medium three times. Transferrin imaging was performed with a confocal microscope at a magnification of 400 **×**.

### Western blot of EEA1 and Rab11

After 12 h treatment with 25 μg/ml 30 nm Ps nanoparticles, RAW 264.7 cells were suspended in PBS by scraping. Protein samples from whole cells were prepared as previously described [8]. Bicinchoninic acid (BCA) protein assay (Beyotime Institute of Biotechnology, Jiangsu, China) was used to determine protein concentrations in samples. Each lane in a 10% polyacrylamide SDS gel was loaded into a 20-μg protein sample, which was then separated by electrophoresis (100 V). The separated proteins were transferred from gels to nitrocellulose transfer membrane (0.2 μm; Whatman, Maidstone, UK) by electroblotting. The membranes were incubated in 5% non-fat milk in TBST (Tris-buffered saline and Tween 20) to block nonspecific binding. The blocked membranes were incubated with a primary antibody against EEA1 (CST, Danvers, MA) or Rab11 (Abcam, Cambridge, UK) (following the manufacturers’ instructions) overnight at 4 °C, then were rinsed three times for 10 min each in TBST. The membrane was then incubated with a corresponding secondary antibody (Santa Cruz Biotechnology, Santa Cruz, CA) for 40 min at room temperature and washed three times for 10 min each in TBST. SuperSignal West Pico (Pierce Biotechnology, Rockford, IL) was used to visualize the protein bands, which were normalized to control bands visualized with a monoclonal anti-ß-GAPDH-IgG antibody (Santa Cruz Biotechnology).

### Statistical analysis

Data were analyzed with SPSS ver. 19.0 software (SPSS, Chicago, IL) using one-way analysis of variance (ANOVA). Results were validated by performing at least three independent experiments. The results were expressed as mean value ± standard error of the mean (SEM). Differences were considered significant at *P <* 0.05.

## Results

### Nanoparticles were internalized by cells

**Fig. 1:**
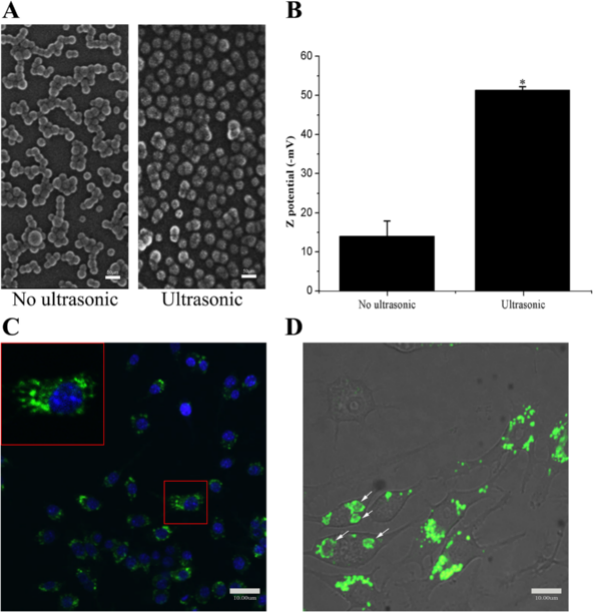
Nanoparticles were internalized by cells. **a**: SEM photographs were 30 nm Ps particles with ultrasonic or not. Utrasonic raised the monodispersion of 30 nm Ps particles obviously. **b**: Zeta potential of 30 nm Ps particels varied from -14 mV to -50 mV with ultrasonic. Potential detector checked the Ps 30 nm particles which were negative on surface. **c**: 30 nm Ps particles labelled with FITC entered the cell and were present in cytoplasm (*red rectangle*). **d**: 30 nm Ps particles gathered in large vesicle-like structures (LVLS) with time going along (*white arrows*)

Images of SEM showed that ultra-sonication increased the number of monodisperse particles (Fig. 1a). DLS assay showed that Zeta potential of the particles varied from −14 mV to −50 mV via sonication of 30 min (Fig. 1b). Characterized Au-NPs with 30 nm diameter was confirmed by SEM and DLS (Additional file 1: Figure S1, A, B). After sonication, the Ps nanoparticles labelled with FITC, was co-cultured with macrophages(RAW264.7 cell line) for 1 h at 4 °C or 37 °C, respectively. Confocal fluorescence microscope showed that the presence of green fluorescence in the cytoplasm of cells cultured at 37 °C (Fig. 1c), but not in those incubated at 4 °C (data not shown). Most of the particles located in vesicles after 1 h (Fig. 1c, a magnified cell was circled by a red line). Large vesicle-like structures (LVLS, white arrow) with fluorescence labels were also present in the cytoplasm (Fig. 1d). Similar phenomenon also presented in this treated cells of A549, HePG-2 and HCT116 (Fig. 3b, c, d).

### Nanoparticles induced the formation of binucleated macrophages

**Fig. 2:**
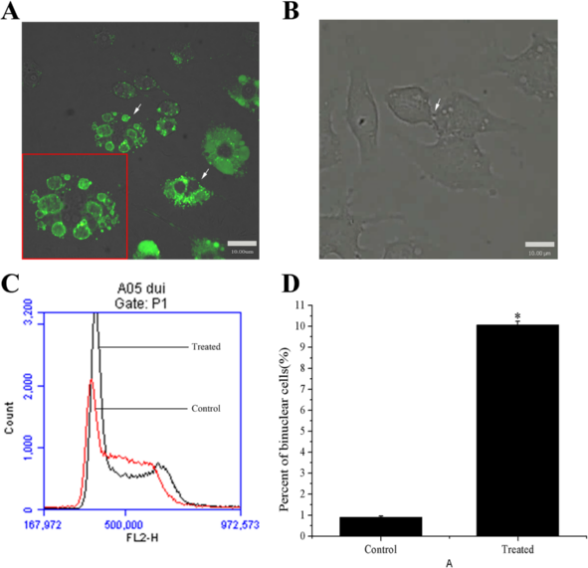
Nanoparticles induced binucleated cells formation. **a**: After co-culture for 12 h, the binuclear cells were present (*red rectangle, white arrows*) and LVLS generated in cytoplasm. A magnified image of binucleated cell showed the 30 nm Ps particles bound on the inner surface of the LVLS. **b**: Video of the process of binuclear cell formation (*white arrow*). **c**: Flow cytometer assay indicated that 30 nm Ps particles increased the percent of binuclear cells (*black line* was treated cell and *red line* was control cell). **d**: The percent of binuclear cells reached 9.97 % in treated cells and was 0.83 % in control cells. The difference of the percent of binuclear cells in treated cell and control cell was significance (*p <* 0.05)


Under different concentrations of 30 nm Ps nanoparticles, varied viability of RAW264.7 cell line was detected using CCK-8 Kit. The varied viability of cells with treated time extension (24 h to 48 h) didn’t show statistically significant compared to the control under different concentrations, but 50ug/ml and the co-culturing time of 48 h depressed the viability of cells clearly. The rate of the binucleated macrophages was the highest under the concentration of 25 μg/ml for 24 h (Additional file 1: Figure S2, A, B). Whereafter, formation of binucleated macrophages was observed by confocal laser scanning microscope after 12 h cultured in 25 μg/ml nanoparticle-containing medium. LVLS outlined with green fluorescence persisted in the cytoplasm, even when two nuclei were visible in the cell (Fig. 2a, red rectangle, white arrow). A movie was recorded by the confocal laser scanning microscope over a 60-min period (1 frame/min) and showed that cytokinesis failed before the formation of a binucleated cell (Fig. 2b, white arrow, Additional file 2: Movie 1). Utilizing flow cytometry and cell count, 9.97% binucleated cells were detected by treatment for 24 h and 0.83% in control cells (Fig. 2c, d). The rate of binucleated cells was expressed as the mean ± standard error of the mean value determined by SEM. The increasing rate was statistically significant compared to the control (*P <* 0.05).

Movie of failing cytokinesis.(AVI 5kb)


### Influence of 30 nm Ps particles on human tumor cell lines

**Fig. 3:**
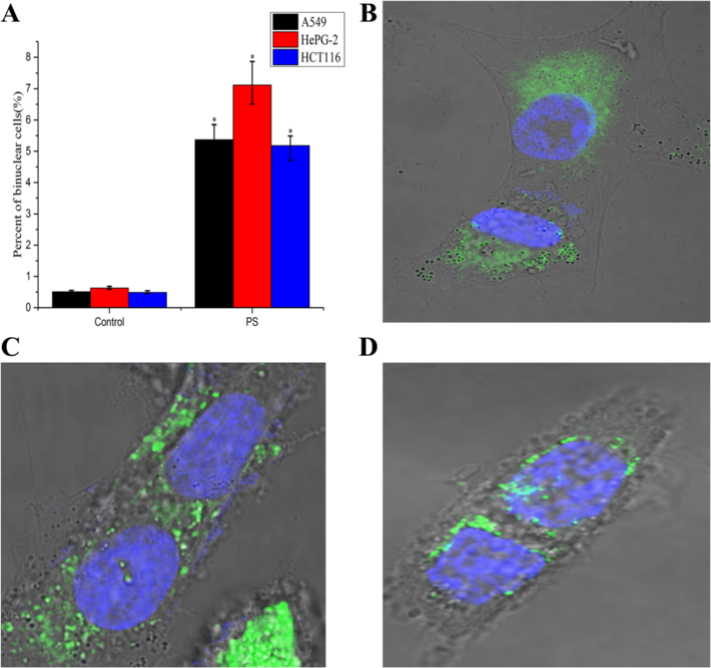
Influence of 30 nm Ps particles on human tumor cells. **a**, binuclear A549. **b**, binuclear HePG-2 **c**: binuclear HCT116. In these binucleated cells, vesicles with green fluorescence of Ps nanoparticles presented in cytoplasm. **d**: the percent of binuclear A549, HePG-2 and HCT116 cells were 5.37 %, 7.12 % and 5.18 % in treated cells to 0.51 %, 0.63 % and 0.49 % in control respectively. The difference of the percent of binuclear cells in treated cell and control cell was significance (*p <* 0.05)

For detecting and confirming of the phenomenon, A549, HePG-2 and HCT116 cell lines were selected to repeat the test. Markedly raised percent of binucleated cells in these treated cells(Fig. 3a,) was detected and confirmed. Green fluorescent vesicles also presented in the cytoplasm of these binucleated cells (Fig. 3b, c, d). However, the rates of binucleated cells in cancer cell lines were lower than in macrophage. Furtherly, the rates of binucleated cells in these cell lines treated by 30 nm Au-NPs (1.575 ng/ml) were calculated. The rates of binucleated cells were also higher in treated cells than control cells (Additional file 1: Figure S4 A). Under the working dose, statistically significant difference of viabilities of treated cells compared to the control wasn’t detected (Additional file 1: Figure S3 B).

### Intracellular transport and distribution of the Ps nanoparticles

**Fig. 4:**
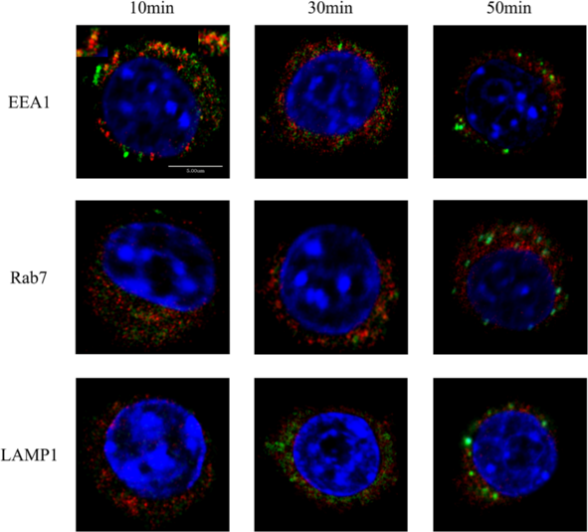
Intracellular transport and distribution of the nanoparticles. EEA1: The co-locations (*yellow*) of EEA1 (*red*) and 30 nm Ps particles (*green*) were present at 10 min, the yellow spots were magnified at right and left superior corners. The co-locations decreased at 30 min, there was hardly co-location and the LVLS generated at 50 min. Rab7: Rab7 co-located hardly with 30 nm Ps particles at 10 min, 30 min and 50 min, the LVLS were also present in the cell at 50 min. LAMP-1: LAMP-1 didn’t co-locate with 30 nm Ps particles from 10 min to 50 min. The co-locations of EEA1 and 30 nm Ps particles at 10 min indicated that the particles entered the cell by endocytic transport. Following that, the particles didn’t co-locate with Rab7 and LAMP-1. That indicated that the particles were not transported through late endosome to lysosome. It meant that the 30 nm Ps particles induced the LVLS formation in early endosome

For detecting the transport of the internalized particles, we tracked existence of particle transport vesicles in the early endosome, later endosome and lysosome in macrophages. RAW264.7 cells were cultured in the particle-containing medium for 10, 30 and 50 min, then rinsed by 0.01 M PBS and labelled EEA1, Rab7 and LAMP-1(markers of early endosome, later endosome and lysosome) with immunofluorescence. Images showed that red fluorescence of EEA1 and green fluorescence of particles were co-localized and yellow spots were already present in the cell at 10 min. After 30 min, the yellow spots disappeared and enlarged green fluorescent flecks present (EEA1, 30 and 50 min). In Fig. 4 (Rab7), labels of Rab 7(red) and the particles (green) weren’t co-localized in cells from 10 min to 50 min. The particles weren’t transported to lysosomes either, because the green fluorescence of particle transport vesicles and the red fluorescence of LAMP-1 weren’t co-localized in cells from 10 min to 50 min (Fig. 4(LAMP-1)).

### Interference with membrane vesicles distribution

**Fig. 5:**
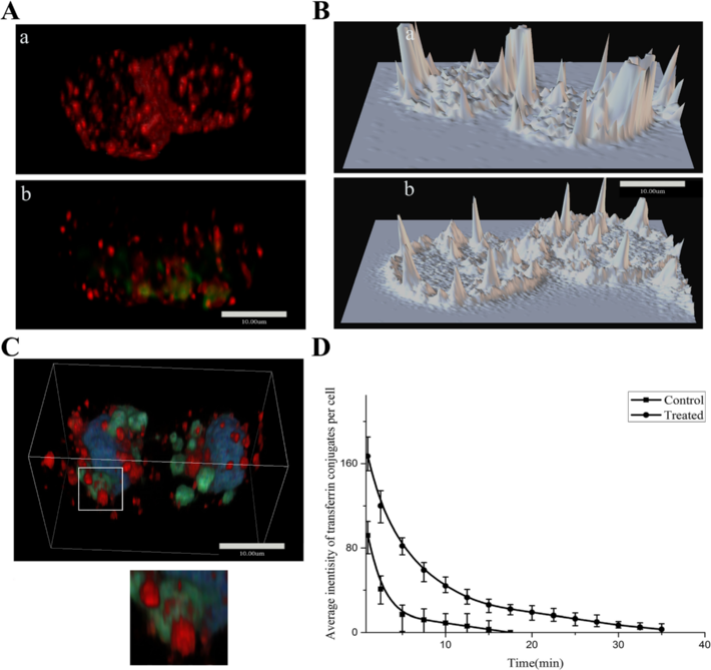
Interference with membrane vesicles distribution. **a**: 3D-reconstruction of control cell and treated cell, red spots of transferrin were present in control (*a*), red spots and green spots of Ps particles were present in treated cell (*b*). The red spots of transferrin accumulated at midbody in control, but were short in treated cell. **b**: Surface Plot of transferrin in control (*a*) and treated cell (*b*), the fluorescence of transferrin distributed and gathered at midbody and poles regions of control cell. But in treated cell, the transferrin dispersed in cytoplasm randomly. **c**. Transferrin located closely to the LVLS contained 30 nm Ps particles (*green*) in 3D space. A magnified image showed the close tethers of transferrin vesicles and LVLS. **d**: The returning time of transferrin from cytoplasm to cell surface in treated cell delayed obviously. The red fluorescent intensity reduction in cytoplasm of control cell was faster than that in treated cell. The t_1/2_ of control cells was 2.5 min and the t_1/2_ of treated cells was 4.9 min. The transferrin vesicles were tethered by LVLS contained 30 nm Ps particles

To visualize the recycling of membrane vesicles through the endosomes during cell mitosis, labeled transferrin conjugates were utilized to trace the endosomes in cells after being treated for 12 h. Confocal fluorescent image showed that red fluorescence flecks of transferrin conjugates in the endosome transferred preferentially closely to midbody region in the control cell (Fig. 5A(a)). The labels didn’t accumulate at the midbody region in treated cell (Fig. 5A(b)). A 3D model was constructed by acquiring a series of Z-axis slices of the confocal fluorescent cell image (Fig. 5B). This surface plot showed localized fluorescence intensity of transferrin positive vesicles in the 3D model. Compared with treated cell (Fig. 5B(b)), the fluorescence intensity of transferrin positive vesicles at the midbody region and the polar region of control cell was higher(Fig. 5B(a)). 3D magnified figure shows that the transferrin positive vesicles preferred locating close to the LVLS (Fig. 5C, a magnified image comes from the region of interest marked by white lines). The efflux of transferrin conjugates from treated cells was slower compared to the control (Fig. 5D). The time necessary for the transferrin to return from the cytoplasm to the cell surface is delayed in treated cells. Half-life time-points (t_1/2_, the time-point when the fluorescence intensity reduced to half of the initial value) of transferrin conjugates effluxes were measured. The t_1/2_ of control cells was 2.5 min and the t_1/2_ of treated cells was 4.9 min.

### Expression and distribution of Rab11 and EEA1

**Fig. 6:**
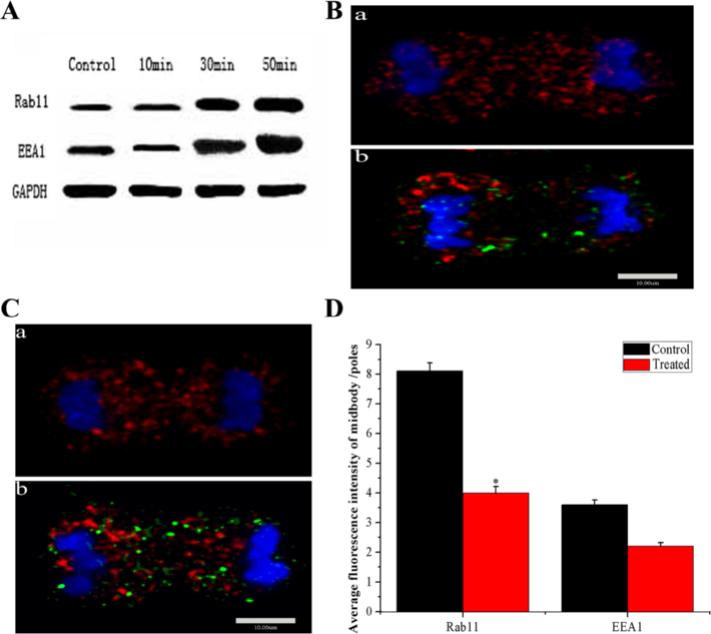
Expressions and distributions of Rab11 and EEA1. **a**: Western blotting revealed that the amount of proteins of Rab11 and EEA1 increased along the co-culturing time (from 10 min, 30 min to 50 min). **b**: (*a*) Red fluorescent spots of Rab11 accumulated at the midbody in control cell of mitosis telophase, (*b*) the red fluorescent spots accumulated to large clusters beside the midbody in treated cell of mitosis telophase. **c**: (*a*) Red fluorescent spots of EEA1 mainly focused at the midbody in control cell of mitosis telophase, (*b*) the red fluorescent spots were less at the midbody and accumulated to large clusters at the poles of treated cell of mitosis telophase. **d**: Fluorescence intensities of Rab11 and EEA1 at midbody were lower obviously after co-cultured with 30 nm Ps particles. Comparted with control, the difference of Rab11 was significance (*p <* 0.05) and EEA1 was without significance in treated cell. Distributions of these key regulators (EEA1 and Rab11) which regulate the organizing of contractile ring and cytokinesis were disturbed obviously

The expressions of Rab11 and EEA1 were detected by western blotting. Compared to the control, gray levels of Rab11 and EEA1 bands (Fig. 6A) were increased in treated cells with extended time of treatment (10, 30, 50 min). Labeled vesicles of Rab11 (red) and EEA1 (red) in cell telophase were traced by confocal microscopy. More labeled vesicles of EEA1(Fig. 6B(a)) and Rab11(Fig. 6C(a)) with higher fluorescent intensities were found in the midbody regions of control cell than in treated cell (Fig. 6B(b).C(b)), which was also confirmed by Meta Imaging Series Software (Molecular Devices, Inc. USA) (Fig. 6D).

## Discussion

Viabilities of RAW264.7 cells, under varied concentrations of Ps nanoparticles, weren’t statistically significant compared to the control after being treated for 24 and 48 h, but the rate of binucleated cells was the highest under the concentration of 25ug/ml for 24 h (Additional file 1: Figure S2, A, B) (*p <* 0.05). A549, HePG-2 and HCT116 cell lines treated by 30 nm Ps nanoparticles and 30 nm Au-NPs respectively also showed statistically significant higher rates of binucleated cells compared to the control (Additional file 1: Figure S4(a)). Therefore, we investigated how the nanoparticles induced binucleated cells.

The uptake of anionic nanoparticles by cells is a two-step process [7, 8], particles are initially bound on the outer cell membrane, then subsequently internalized. While binding can occur at any temperature, internalization can’t occur at 4 °C because that the low energy level at this temperature prevents endocytic uptake.

Confocal microscopy detected green fluorescence in the cytoplasm of RAW264.7 cells cultured at 37 °C, but not in those incubated at 4 °C. This suggested that most of the particles had been internalized via endocytic pathway. However, a previous study showed that approximately half of the Ps particles were present in the cytoplasm even though cells were incubated at 4 °C [9]. We have found that the 30 nm Ps particles diluted with fresh medium were internalized by macrophages at 4 °C if they were treated by sonication. However, it has been observed that the endocytic uptake of Ps particles of 50 and 100 nm diameters was still blocked at 4 °C even after sonication. Both dynamic light scattering (DLS) and SEM revealed that the particles size and size distribution varied depending on their treatment (Fig. 1a, b). Therefore, we used ultrasonic treatment for 30 min to increase the mono-dispersity of 30 nm particles, which allowed them to directly pass through the plasma membrane [9]. The aggregated particles were also taken into macrophages, but by endocytosis. An unexpected result is that the particle’s zeta potential changed as the result of sonication from −14 mV for the aggregates to −50 mV for the well dispersed primary particles. This implies that the aggregated particles are unstable. It is important to note that the early endosome lumen generally appears in the setting of ATP-dependent acidification. Under those conditions, uptake of aggregated particles may be dispersed to the organelles. Figure 1c shows that most particles were located in vesicles after cells were co-cultured with particles for 1 h. Large vesicle-like structures (LVLS, white arrow) with green fluorescent labeled particles were also present in the cytoplasm (Figure 1d). In the endocytic pathway, transport vesicles pinch off pieces of the plasma membrane and fuse with early endosomes and with incoming transport vesicles as well as with each other. Rink et al. reported that the formation of larger vesicles is due to homo or heterotypic fusion of early endosomes [10]. Thus, we suggest that LVLS are endosome components that are formed by continuous fusion of early endosome-containing particles with transport vesicles. Varied state of Ps particles in endosome was a dynamic process of thermodynamic stabilization when these particles were transported in cell. Initial state of these particles was aggregation. Big green fluorescent spots consisting of particles appeared in the endosomes (Fig. 1c and d). Then these particles were dispersed and bound on the inner membrane of endosomes due to varied internal microenvironments (Fig. 2a). The binding hold endosome and formed the LVLS (large vesicle like structures).

Furtherly, binucleated cells were observed in 12 h cultures of nanoparticle-containing medium (Fig. 2a, b). We have recorded a video that showed how influence of nanoparticle uptake load in macrophages and eventually to failed cytokinesis and binucleated cell formation(Fig. 2b, Additional file 2: Movie 1). After 24 h, flow cytometry and cell counting revealed the presence of 9.97% and 0.83% binucleated cells in treated and in control cells, respectively (*p <* 0.05) (Fig. 2c,d). Cytokinesis is a very complex and highly orchestrated physical separation of daughter cells. Its completion is dependent on membrane transport and subsequent fusion in midbody region of the cells. The localization of pivotal cytokinesis proteins depends on the traffic of membrane vesicles coming from endosomes. A few researchers have found that deposition of membrane vesicles at the cleavage furrow occurs during cytokinesis [11, 12]. In addition, cytokinesis requires membrane insertion at the midbody region to increase the cellular surface area and allow daughter cells separation. Because plasmalemma synthesis by the Golgi apparatus is blocked in telophase [13, 14], membrane trafficking from the endosome is the primary plasmalemma source for cytokinesis [15, 16]. However, LVLS (marked by green fluorescence in Fig. 2a) persisted in the cytoplasm, even when two nuclei were visible in the cell. Although the morphology of the binucleated cell wasn’t like apoptosis, we still assayed the level of Annexin V conjugates in cells. Compared with the control cells, the Annexin V conjugates in the treated cell didn’t increase (data not shown). The result implied that the LVLS were maintained by the particles packaged in the structures. The LVLS may be in a biologically inert state due to the binding of the particles, and because there was a limited supply of membrane components for cytokinesis, the presence of nanoparticles prevented this process from occurring. In induced and formed binucleated cell of A549, HePG-2 and HCT116, green fluorescent vesicles were also in cytoplasm (Fig. 3b, c, d).

Nascent transport vesicles rapidly fuse with early endosomes, late endosomes, and eventually with lysosomes. We examined all three structures to delineate the transport pathway of the internalized particles. The tethering protein EEA1 is localized exclusively at early endosomes and is used to target intracellular organelles [17]. EEA1 also has an important role in determining endosome fusion efficiency [18]. We examined EEA1 redistribution by co-culturing Ps nanoparticles with RAW264.7 cells for 10 min (Fig. 4, EEA1, 10 min). In these confocal images we found yellow spots in the cells where red EEA1 fluorescence and green particle fluorescence merged. After 30 min, the yellow spots disappeared, but enlarged green fluorescent blotches were present even though the particle containing culture medium has been replaced with fresh medium (Fig. 4, EEA1, 50 min). Small GTPases of the Rab family are important vesicular transport regulators located in specific intracellular compartments. Rab7 has been shown to localize to late endosomes of the endocytic pathway [19]. Figure 4 shows that Rab7 (red) and the particles (green) weren’t co-localized in cells from 10 to 50 min (Fig. 4, Rab7, 10, 30 and 50 min). LAMP-1 is a glycoprotein that is a useful lysosomal marker [20]. The green fluorescence of particles didn’t co-localize with the red fluorescence of LAMP-1, indicating that the particles weren’t transported to lysosomes (Fig. 4, LAMP1, 10 30 and 50 min). This phenomenon was persistently in cell with time extended to 120 min (Additional file 1: Figure S6,A,B,C). An interesting phenomenon was that green fluorescence of LVLS persisted for 12 h. Our research has shown that Ps particles (d = 50 and d = 100 nm) can be transported to lysosomes [8]. The presence of LVLS (with a clearly outlined green fluorescence, Fig. 2a, white arrow) in binucleated cells implied that the aggregated particles were dispersed and bound on the inner membrane of these endosomes. This prevented the transport of nanoparticles from early endosomes, and the pathway from late endosome to lysosome was also restricted. Membrane traffic from LVLS was also blocked. Further research is needed to clarify the detailed mechanism of this process.

There are reports that transport vesicles from the endosome provide the source of new membrane; these membrane vesicles are deposited in the midbody to form a cleavage furrow and divide the cell [11, 12]. Boucrot et al. used fluorescently labeled transferrin, a ligand that was specifically taken up by clathrin-based endocytic vesicles, to target membrane vesicles recycled from endosomes during cell mitosis [11]. This method was also utilized in our experiments to trace transferrin vesicles in the treated cells. Confocal fluorescent microscopy showed that vesicles with red fluorescence were preferentially located near the midbody of control cells in telophase (Fig. 5A(a)), but this wasn’t obvious in treated cells (Fig. 5A(b). A reasonable hypothesis is that internalized particles are distributed to the cell midbody during anaphase and telophase if indeed the membrane vesicles from LVLS participate in membrane trafficking for cytokinesis. Figure 5A(b) shows that treated cells lacked green fluorescence in the midbody region in telophase, whereas red labels of transferrin conjugates were also randomly distributed in the cytoplasm of treated cells. Schweitzer et al. reported that transferrin vesicles were transported from the distal endosomal cluster to the intercellular midbody in late mitosis [21]. In our experiment, the phenomenon was also present in control cell (Fig. 5A(b)). Under 3D high magnification, these transferrin-positive vesicles in treated cells weren’t proximal to the midbody, rather, they were near the LVLS (Fig. 5C, A magnified image comes from the region of interest marked by white line). The efflux of transferrin conjugates from the treated cell was slower compared to the control (Fig. 5D). The time for transferrin returning back to the surface membrane of the cell was delayed in treated cells (the t_1/2_ of control cells was 2.5 min and the t_1/2_ of treated cells was 4.9 min). This result implies that the LVLS tether these transferrin-positive vesicles and disturbs the membrane vesicle transport of transferrin during telophase (Additional file 1: Figure S5).

Endosomes transport of Rab11 to the midbody of mitotic cells is necessary for establishing the abscission site. During this process, Rab11 GTPase binds and activates various effector proteins involved in targeting vesicles during mitosis. EEA1 is localized exclusively to early endosomes [18], where it acts as a tethering molecule that couples vesicle docking with SNARE proteins to bring the endosomes physically closer and facilitate their fusion. We assessed Rab11 and EEA1 expressions by western blotting and found that both Rab11 and EEA1 (Fig. 6A) were increased in treated macrophages. Confocal fluorescent microscopy revealed the distributions of these cells with Rab11 and EEA1 label (red) in telophase, there were fewer labeled vesicles were found in the midbody region of treated cells (Fig. 6B(b),6C(b)) compared with control cells. Thus, internalized particles induced high Rab11 and EEA1 expressions, but LVLS with particles disturbed their distributions. Accurate Rab11 localization is critical for the execution of cytokinesis. A series of studies revealed that Rab11-binding proteins FIP3 and FIP4 located to the midbody and simultaneously interacted with Rab11 to promote cytokinesis [22–24]. A reasonable suggestion is that LVLS in nanoparticle-treated cells altered Rab 11 and EEA1 localizations.

The research article assessed the influences of 30 nm Ps particles and 30 nm Au-NPs on the endocytic pathway in RAW264.7, A549, HePG-2, and HCT116 cell lines, through the formation of LVLS, both macrophage(RAW264.7) and human cancer cell lines(A549, HePG-2, HCT116) resulted in increasing percent of binucleated cells. Many papers have reported the formation of binucleated cells by interfering in the endocytic pathway, but the process of internalization of nanoparticles (30 nm Ps particle and 30 nm Au-NPs) through endocytic pathway could lead to the same effect has not been reported intensively. So our work described a new phenomenon of nanoparticles effect on endocytic pathway in different cell lines. On the other side, all of the data supporting our opinion was collected on cell level only and under our laboratory conditions.

## Conclusion

These internalized particles induce fusion of endosome and the formation of LVLS. In these structures, the aggregated particles disperse and bind on the inner endosome membrane depending on the microenvironment. The LVLS block membrane trafficking from the LVLS and the endosome, limit the amount of membrane components available, and alter the localization of pivotal proteins required for cytokinesis. Importantly, we have found that nanoparticle-treated cells have difficulty completing this important process because of limited membrane trafficking.

## Additional files

Additional file 1: Supplemental data. (DOCX 22634 kb)

## Abbreviations

BCA: Bicinchoninic acid
BSA: Bovine serum albumin
CCK-8: Cell counting kit-8
DMEM: Dulbecco’s minimal essential media
EEA1: Early endosomal antigen-1
FBS: Fetal bovine serum
LVLS: Large vesicle-like structures
Rab: Ras-related protein
TBST: Tris-buffered saline and tween 20
