# Supplementary material for: Endocytosed nanoparticles hold endosomes and stimulate binucleated cells formation
Source: Part Fibre Toxicol. 2016 Nov 29;13:63. doi: 10.1186/s12989-016-0173-1 (PMC5127043; doi:10.1186/s12989-016-0173-1)
Supplement: Additional file 1: — Supplemental data. (DOCX 22634 kb) [file 12989_2016_173_MOESM1_ESM.docx]

**Supplementary material**

**SEM and DLS measurement of Au-NPs**

**
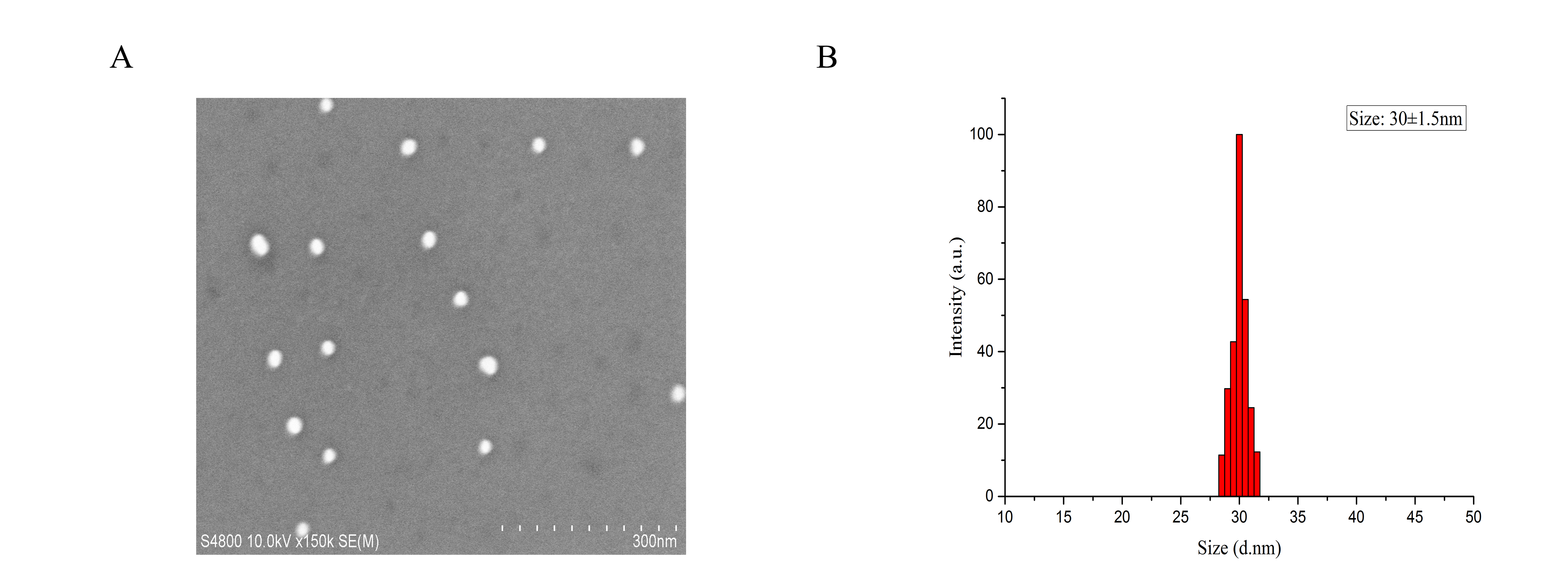
**

**Figure.S1 A**:SEM photograph of the used Au-NPs without metal-spraying, indicated that the diameters of the particles were uniform and monodispersed, most of the Au-NPs shown in the photograph concentrated to the diameter of 30nm. The Synthesis was performed by citric acid reduction (1%auric chloride acid/1% sodium citrate in 100ml deionized water under boiling for 30min). **B**: DLS charactered the distribution of Au-NPs diameter, the diameter of the particles concentrated at 30nm relatively. The diameters of the Au-NPs varied between 30±1.5nm, the distribution of the Au-NPs diameters was concentrated, the diameters of the Au-NPs was at the same level of the 30nm Ps particles and satisfied the experiment requirements.

**CCK-8 measured cell viability of different concentrations of PS nanoparticle**

**
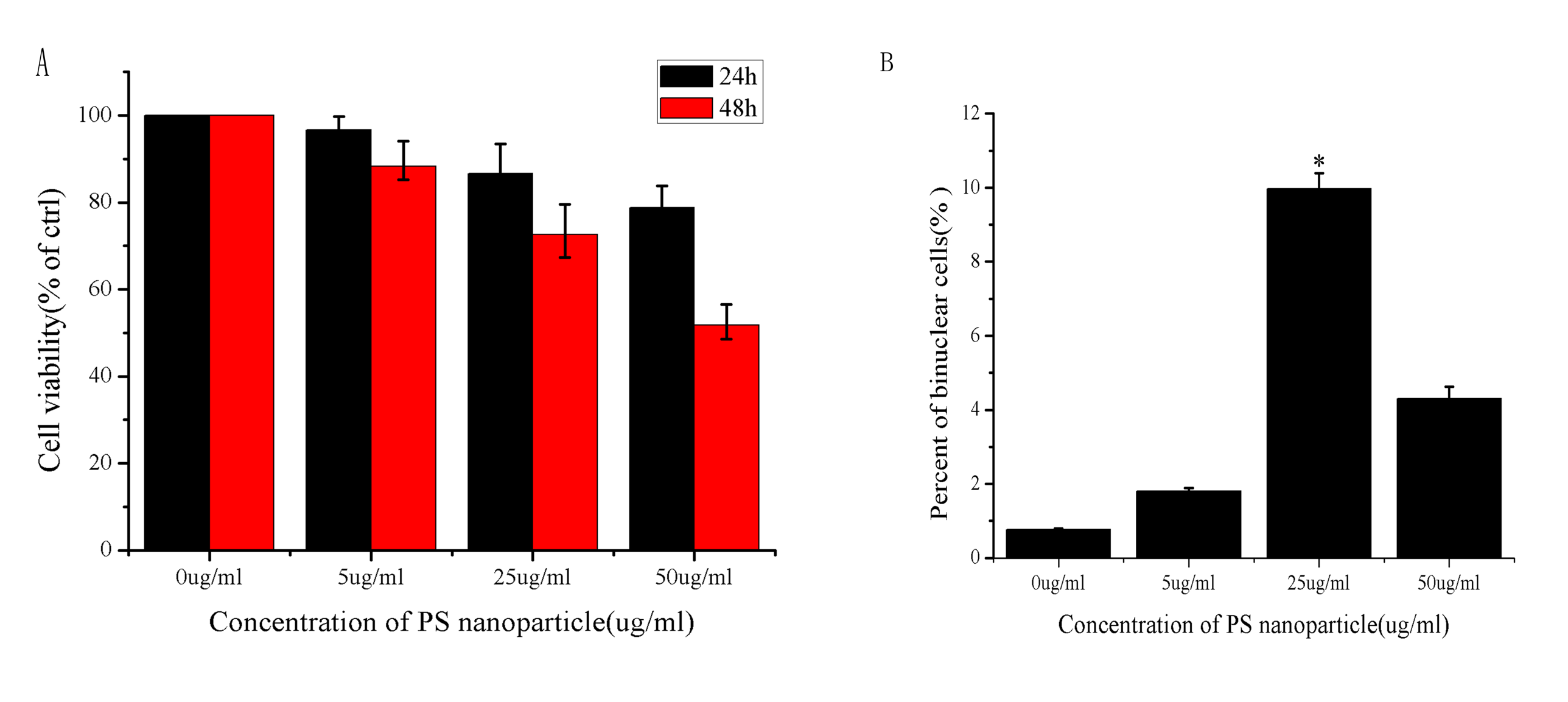
**

**Figure.S2 A**, CCK-8 assay indicated that the variation of viability of cells with the time going along(24 and 48h) or concentration of 30nm PS nanoparticle increasing (5, 25 and 50µg/ml). The viability of cell with treated time extension (12h to 48h) did no show statistically significant compared to the control in varied 30nm particle concentation. But, 50ug/ml and the co-culturing time of 48h depressed the viability of cells. The viability of cells decreasd nearly to 50% accoding to the control. **B**, The rate of binucleated cells was highest under the concentration of 25ug/ml at 24h. At the same time point, the cell viability keeped above 50% according to the control, so we chose 25ug/ml as experiment concentration[1].

**CCK-8 measured cell viability of 30nm Ps nanoparticles on human tumor cell lines.**

**

**

**Figure.S3** CCK-8 assay indicated that the viability of 30nm Ps particles on human tumor cell lines (A549, HePG-2, HCT116) with the time going along(48h) at the concentration of 25µg/ml. The viability of cell with treated time extension (12h to 48h) did no show statistically significant compared to the control in varied 30nm particle concentation did no show statistically significant compared to the control.

**Movie of binucleated cell formation(Movie.1)**.

**Binucleated cell statistics and cytotoxicology of 30nm Au-NPs on different cell lines.**

**
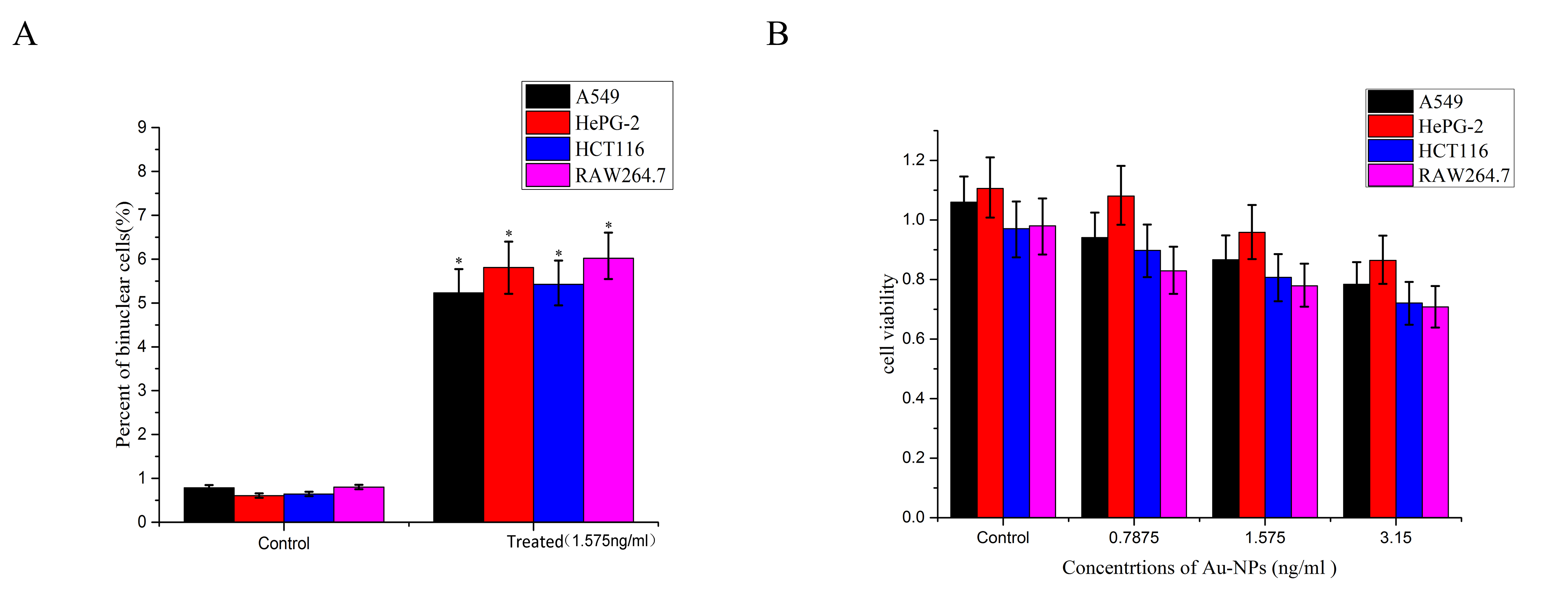
**

**Figure.S4 A,** the treatment of Au-NPs raised percent of binucleated cells in A549, HePG-2, HCT116 and RAW264.7 cell lines(5.232, 5.811,5.426, 6.023 in treated cells to 0.787, 0.608, 0.644, 0.802 in control). The difference were considered significance(P<0.05). **B**,CCK-8 assay indicated that the viability of A549, HePG-2, HCT116 and RAW264.7 didn`t decrease obviously at 0.7875,1.575 and 3.15ng/ml Au-NPs. So the toxicology of different concentrations (0.7875,1.575 and 3.15ng/ml) of Au-NPs were not obviously, we chose the concentration of 1.575ng/ml as our experiment concentration.

**Transferrin rate of circulation detection.**

In treated cells, the transferrin fluorescence intensity at midbody was obviously higher than control(Figure. S5A). Comparted with control, the ratio of fluorescence intensity of transferrin internalized the cell to that on surface of the cell is also higher. The LVLS were next to transferrin closely in space, and hold the endosomes in recycle. So the LVLS interfered into the transferrin recycling.

Cells adherent to round coverslips were preincubated with 25 µg/ml particles in DMEM/F-12 for 12 h at 37℃. The medium was aspirated and discarded, then cells were washed three times with PBS followed by incubation on ice for 20 min in DMEM/F-12 medium containing 20 mM glucose and 1% bovine serum albumin (BSA). Next, we added transferrin-Alexa Fluor 555 (Thermo Fisher Scientific, Waltham, MA) to the medium at a final concentration of 25 µg/ml and incubated at 37°C for 20 minutes. Afterward, cells were washed with fresh medium three times. Transferrin imaging was performed with a confocal laser scanning microscope at a magnification of 400×. The transferrin fluorescence on the cell surface was calculated through taking the slice of the cell surface. The transferrin fluorescence internalized the cell was calculated through summarizing fluorescence of all Z-axis slices about the space internalized the cell except the slice of the cell surface. The incement of Z-axis was 0.25um[2].


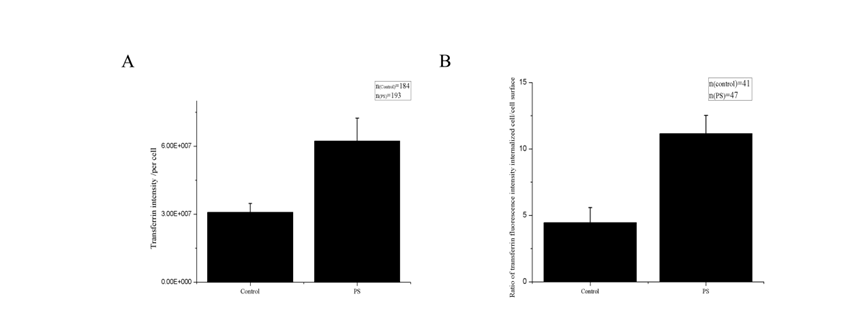


**Figure.S5 A**: averge transferrin fluorescence intensity per cell, the treated cell was obviously higher than the control cell. **B**: the ratio of transferrin fluorescence intensity internalized the cell to that on cell surface is also higher in treated cells than that in control.

**LVLS were within endocytic system and formed after 30-nm PS particles separated from early endosome but didn`t enter late endosome or lysosome.**


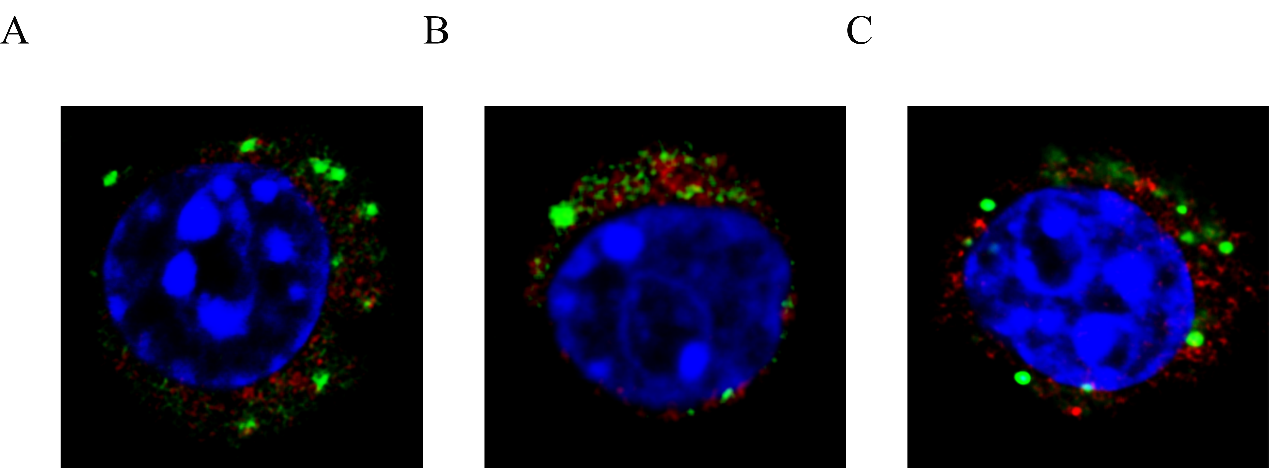


**Figure.S6** After co-location for 10min, 30nm PS particles separated from EEA1, and didn`t co-located until 120min(Figure. S6A). On the other hand, Rab7 and LAMP-1 didn`t co-locate with the particles within 120min(Figure.S6B,C). So, these data illustrated that the 30nm Ps particles entered early endosome at the 10^th^ min, but didn`t enter late endosome and lysosome. After separation from early endosome, the LVLS generated, and the particles accumulated in LVLS.

**Ps particles reduced the proliferation rate of RAW264.7 cell line**

We measured cell numbers at different timepionts(RAW264.7 cell line) with flow cytometer, and drew the proliferation curves. The timepoints checked were 0,12,24,36 and 48h.





**Figure. S7** the proliferation rate of control was more than the treated during 48h, during to the influence of Ps particles on cytokinesis, a percent of binucleated cell was present in cells, the binucleated cells couldn`t perform cell-cycle again, so the proliferation rate decreased obviously in treated cells.

1. Zhang M, et al., Variation in the internalization of differently sized nanoparticles induces different DNA-damaging effects on a macrophage cell line. Archives of Toxicology. 2011;85(12):1575-1588.

2. Boucrot E,Kirchhausen T, Endosomal recycling controls plasma membrane area during mitosis. Proceedings of the National Academy of Sciences of the United States of America. 2007;104(19):7939-7944.
